# Memory-Efficient Training for Fully Unrolled Deep Learned PET Image Reconstruction with Iteration-Dependent Targets

**DOI:** 10.1109/TRPMS.2021.3101947

**Published:** 2021-08-02

**Authors:** Guillaume Corda-D’Incan, Julia A. Schnabel, Andrew J. Reader

**Affiliations:** School of Biomedical Engineering and Imaging Sciences, Department of Biomedical Engineering, King’s College London, St. Thomas’ Hospital, London, UK

**Keywords:** PET reconstruction, deep learning, model-based image reconstruction

## Abstract

We propose a new version of the forward-backward splitting expectation-maximisation network (FBSEM-Net) along with a new memory-efficient training method enabling the training of fully unrolled implementations of 3D FBSEM-Net. FBSEM-Net unfolds the maximum *a posteriori* expectation-maximisation algorithm and replaces the regularisation step by a residual convolutional neural network. Both the gradient of the prior and the regularisation strength are learned from training data. In this new implementation, three modifications of the original framework are included. First, iteration-dependent networks are used to have a customised regularisation at each iteration. Second, iteration-dependent targets and losses are introduced so that the regularised reconstruction matches the reconstruction of noise-free data at every iteration. Third, sequential training is performed, making training of large unrolled networks far more memory efficient and feasible. Since sequential training permits unrolling a high number of iterations, there is no need for artificial use of the regularisation step as a leapfrogging acceleration. The results obtained on 2D and 3D simulated data show that FBSEM-Net using iteration-dependent targets and losses improves the consistency in the optimisation of the network parameters over different training runs. We also found that using iteration-dependent targets increases the generalisation capabilities of the network. Furthermore, unrolled networks using iteration-dependent regularisation allowed a slight reduction in reconstruction error compared to using a fixed regularisation network at each iteration. Finally, we demonstrate that sequential training successfully addresses potentially serious memory issues during the training of deep unrolled networks. In particular, it enables the training of 3D fully unrolled FBSEM-Net, not previously feasible, by reducing the memory usage by up to 98% compared to a conventional end-to-end training. We also note that the truncation of the backpropagation (due to sequential training) does not notably impact the network’s performance compared to conventional training with a full backpropagation through the entire network.

## I Introduction

Positron emission tomography (PET) provides crucial functional information allowing to detect abnormalities undetectable by other imaging modalities. However, PET measured data often suffers from high noise levels due to detector sensitivities or numerous physical processes limiting the number of back-to-back photon pairs collected by the scanner. Besides, high injected radiotracer dose can expose patients as well as technicians to health risks, leading to the need for dose reductions further degrading the measured data quality. The classic maximum likelihood expectation-maximisation (MLEM) algorithm [1] or its variant, the ordered-subsets expectation-maximisation (OSEM) algorithm [2] used for reconstructing PET images in routine scans tend to fit the noise contained in the data. They deliver 'night sky' reconstructions, hence the need for regularisation. The latter is currently realised by terminating the MLEM algorithm before it reaches convergence or by applying post-smoothing on the reconstructed images. Regularisation can also be achieved by introducing prior information in the reconstruction process. Recovering an unknown image is therefore done by estimating the maximum *a posteriori* (MAP) using the expectation-maximisation algorithm. Some priors only use the PET image such as the quadratic prior or total variation (TV). Nonetheless, depending on the data and/or the regularisation strength chosen, these priors show their limitations as they can provide very unnatural looking images (over-smoothing or staircase artifacts). Other priors have been designed based on the idea that neighbouring voxels should have similar intensity values whereas distant voxels should not. Local, initially, then non-local edge-preserving priors [5], [6] have successfully provided robust regularisers. In case of extreme levels of noise, the risk of missing boundaries remains present. An other approach to improve PET image quality is to use anatomical information obtained from magnetic resonance imaging (MRI) [7], [8], [9] or computed tomography (CT). These high resolution images can help in guiding the PET reconstruction to recover lost boundary information. In spite of years of research, all the proposed regularisation methods remained hand-crafted and designed to match some desirable mathematical properties or impose beliefs that we have.

Recently, deep learning methods opened a new area of research for image computing, demonstrating improved performance compared to state-of-the-art conventional algorithms [18]. Deep neural networks can roughly be used in four ways for medical image reconstruction: i) for pre-processing of the measured data [16], ii) for post-processing of the reconstructed images [17], iii) to learn the entire reconstruction process [10], [11], iv) to complement conventional reconstruction algorithms. The focus here will be on the latter category, namely physics-informed deep learning. In this framework, the strengths of robust statistical noise models are combined with neural networks. Conventional iterative reconstruction algorithms are unrolled and neural networks are incorporated to learn the parts of the reconstruction that we are not certain of, such as the prior form or the regularisation strength. Three main frameworks have been proposed for PET reconstruction. The method from Gong et al. [12] named EM-Net and later updated in MAPEM-Net is a method based on the alternating direction method of multipliers (ADMM) algorithm. They alternatively perform two MAP-EM updates followed by a denoising step using a neural network. The resulting denoised image is thereafter used as a prior image for the following two MAP-EM updates. In MAPEM-Net, a U-Net structure is selected for the neural networks and eight unrolled modules are used. The networks are trained end-to-end which might lead to potential memory issues.

Lim et al. [13] proposed unrolling a block coordinate descent (BCD) algorithm. They adapted the so-called BCD-Net [21], first proposed for MR reconstruction, to PET reconstruction. In this framework, similarly to MAPEM-Net, they alternate between a given number of reconstruction steps and denoising steps. The architecture of the network for their iterative NN (INN) is a simple 2-layer convolutional encoder-decoder (CED) whose activations (soft-thresholding operations) are learned during the training process. The network is initialised with an image obtained after a few EM updates before alternating between a pass through a convolutional image denoising (CID) module and an image reconstruction module. The framework is designed such that it is possible to conduct multiple passes through the image reconstruction module (or conduct an entire reconstruction) before to pass once into the CID module. Because the CID modules do not contain any time-consuming forward or back projectors, the INN training is considerably sped up compared to other unrolled methods. Additionally, the memory requirements are lower as the intermediate reconstruction results do not have to be stored in memory. Nonetheless, just like MAPEM-Net, a reduced number of unrolled modules is used compared to the number of iterations used for the reconstruction of the targets, which introduces what we call leapfrogging. The problem with such techniques is that regularisers can learn object details and thus become more than simple learned regularisers, putting at risk their generalisation abilities. These networks can precipitate the convergence of the conventional algorithm.

The third framework is the forward-backward splitting expectation-maximisation (FBSEM) network [4]. FBSEM-Net unfolds the MAP-EM algorithm and replaces the regularisation step by a residual convolutional neural network to learn both the gradient of the prior and the regularisation strength. However, some issues arise from its original implementation that we improve upon in this work to make it more practical and offer a slight gain in performance. Specifically, we propose using iteration-dependent networks and iteration-dependent targets/losses such as in [20]. We will be referring to this new version as FBSEM-IS-Net (FBSEM iteration-specific). To address potential memory issues occurring during the training of deep unrolled networks such as FBSEM-Net, we also investigate the impact of training all the modules of the unrolled network independently rather than training the network in an end-to-end fashion. FBSEM-IS-Net has 60 times more trainable parameters than in the original version. Its generalisation abilities are assessed on piece-wise constant phantoms. This method, while applied to the FBSEM framework, can be extended to enable or facilitate the training of any deep unrolled network. The main contributions of this work are first to increase the generalisation capabilities of deep unrolled networks using iteration-dependent targets and losses by constraining the regularisers to be pure denoisers. Second, this work enables training of unrolled networks for higher numbers of iterations, hence avoiding any artificial acceleration of the reconstruction algorithm by leapfrogging. The sequential training proposed allows the training of a fully unrolled version of 3D FBSEM-Net for the typical numbers of iterations that would be normally chosen for MAP-EM methods.

The structure of this article is as follows. Section II reviews the basic principles of model-based PET image reconstruction, describes the FBSEM framework and develops the new version proposed. Section III provides information on the data used along with the process to generate it, gives implementation details and describes the various reference methods compared with our proposed FBSEM-IS-Net. Section IV presents how the different methods perform on 2D and 3D simulated test data. Finally, Section V and VI conclude and discuss on potential improvements of the proposed method.

## II Theory and Methods

### A Model-based PET reconstruction

Model-based image reconstruction (MBIR) methods seek to iteratively estimate an image **x** ∈ ℝ^*N*^ from noisy measurements **y** ∈ ℝ^*M*^, *N* being the number of voxels and *M* the number of sinogram bins. In PET reconstruction, the *y_i_* are assumed to be drawn from a Poisson distribution and their mean is modelled by

(1) $$\bar{\text{y}}=\text{Px}+\text{r}+\text{s}$$

with **P** ∈ ℝ^*M*×*N*^ the system matrix and **r** + **s** ∈ ℝ^*M*×1^ the mean randoms and scatters. The image **x** is estimated by solving the following ML problem:

(2) $$\hat{\text{x}}=\underset{\text{x}}{\text{argmax}}L\left(\text{x}|\text{y}\right)$$

where *L* is defined as the Poisson log-likelihood

(3) $$L\left(\text{x}|\text{y}\right)=\sum_{i}y_{i}\text{log}\left(\bar{y_{i}}\right)-\left(\bar{y_{i}}+\log\left(y_{i}!\right)\right)$$

However, the MLEM algorithm tends to fit the noise contained in the measured data to produce noisy estimates. A solution to circumvent this problem is to add a constraint into the objective function imposing prior knowledge we have about **x**. We are no longer seeking to estimate the ML but the MAP using the EM algorithm:

(4) $$\hat{\text{x}}=\underset{\text{x}}{\text{argmax}}L\left(\text{x}|\text{y}\right)-\beta R\left(\text{x}\right)$$

where *R* is a penalty term that enforces **x** to be in the set of realistic images and *β* is the regularisation strength.

Notwithstanding the quantity and diversity of priors proposed in the literature, the majority are designed to match specific requirements or introduce beliefs that we have about **x** but are unlikely to be optimal. A recent trend has been to use learned priors.

### B FBSEM-Net

The optimisation transfer technique can be used to solve Eq. (4) under the condition that a differentiable, separable and convex surrogate is used for the prior *R*. In the FBSEM-Net framework, the forward-backward splitting (FBS) algorithm [14] is used. By replacing the projection operator by a proximal mapping, the FBS algorithm offers a generalisation of the projected gradient descent algorithm. The optimisation problem can therefore be rewritten as follows:

(5) $${\text{x}}^{\left(n\right)}=\underset{\text{x}}{\text{argmax}}\;L\left(\text{x}|\text{y}\right)-\frac{1}{2\gamma }{\left\|\text{x}-{\text{x}}_{Reg}^{\left(n\right)}\right\|}^{2}$$

(6) $${\text{x}}_{\mathrm{Reg}}^{\left(n\right)}={\text{x}}^{\left(n-1\right)}-\gamma \beta \nabla R\left({\text{x}}^{\left(n-1\right)}\right)$$

In Eq. (5) a proximal mapping is performed, while in Eq. (6) a gradient descent update is performed with a step size of *γ*. The trade-off between data consistency with the likelihood *L* and the discrepancy between **x** and ${\text{x}}_{\text{Reg}}^{\left(n\right)}$ is controlled by $\frac{1}{2\gamma }.$ A surrogate can be defined for *L* [15] and Eq. (5) can be reformulated:

(7) $${\text{x}}^{\left(n\right)}=\underset{\text{x}}{\text{argmax}}{\sum_{j}x_{j,EM}^{\left(n\right)}ln\left(x_{j}\right)-x_{j}-\frac{1}{2\gamma s_{j}}\left(x_{j}-x_{j,Reg}^{\left(n\right)}\right)}^{2}$$

with

(8) $$x_{j,EM}^{\left(n,m\right)}=\frac{x_{j}^{\left(n-1,m\right)}}{s_{j}}\sum_{i}p_{ij}\frac{y_{i}}{\sum_{k}p_{ik}x_{k}^{\left(n-1,m\right)}+r_{i}+s_{i}}$$

and *s_j_* = ∑_*i*_ *p_ij_*.

Setting the derivative of Eq. (7) to zero gives the following closed-form solution:

(9) $$x_{j}^{\left(n,m\right)}=\frac{2x_{j,EM}^{\left(n,m\right)}}{1-\frac{x_{j,\text{Reg}}^{\left(n,m\right)}}{\gamma s_{j}}\sqrt{(1-\frac{x_{j,\text{Reg}}^{\left(n,m\right)}}{\gamma s_{j}}{)}^{2}+\frac{x_{j,EM}^{\left(n,m\right)}}{\gamma s_{j}}}}$$

In FBSEM-Net, a convolutional neural network (CNN) is incorporated in the regularisation step Eq. (6) to learn the gradient of the prior and the regularisaton strength. Therefore, no hyperparameter has to be set by the user. The resulting algorithm is composed of 3 steps: i) data consistency update, ii) regularisation update, iii) fusion of the two resulting images (see Algorithm 1). We refer to one iteration of the unrolled algorithm (the completion of these three steps) as a module. The same set of parameters ***θ*** for the CNN is shared across all the modules. FBSEM-Net has shown improved results for PET reconstruction compared to state-of-the-art methods. Nonetheless, some issues arise from its original implementation. First, the training of FBSEM-Net has massive memory requirements. Unrolling 60 modules becomes challenging if not impossible when working with 3D data as all the intermediate images have to be stored in memory. Second, the use of a single final loss does neither limit the residual CNN to act as a pure regulariser nor control the output of the intermediate modules. The network could learn object details and artificially accelerate the convergence of the algorithm. It is based on these concerns that we proposed FBSEM-IS-Net along with the sequential training method.

Algorithm 1 **: FBSEM-Net**

**Initialize**: x^(0)^ = **1**, *γ* ∈ [0,1], *N_iters_* = 10, *N_subsets_* = 6

**for** *n* = 1 … *N_iters_* **do**

      **for** *m* = 1 … *N_subsets_* **do**

            $x_{j,OSEM}^{\left(n,m\right)}=\frac{x_{j}^{\left(n-1,m\right)}}{s_{j}}\sum_{i}p_{ij}\frac{y_{i}}{\sum_{k}p_{ik}x_{k}^{\left(n-1,m\right)}+r_{i}+s_{i}}$

            with $s_{j}=\sum_{i}p_{ij}$

            $x_{j,\text{R}\text{eg}}^{\left(n,m\right)}=x_{j}^{\left(n-1,m\right)}-\gamma \beta {ℱ}_{PET}\left({\text{x}}^{\left(n-1,m\right)},\theta \right)$

            $x_{j}^{\left(n,m\right)}=\frac{2x_{j,OSEM}^{\left(n,m\right)}}{1-\frac{x_{j,\text{R}\text{eg}}^{\left(n,m\right)}}{\gamma s_{j}}+\sqrt{\left(1-\frac{x_{j,\text{R}\text{eg}}^{\left(n,m\right)}}{\gamma s_{j}}\right)2+4\frac{x_{j,OSEM}^{\left(n,m\right)}}{\gamma s_{j}}}}$

      **end**

**end**

### C FBSEM-IS-Net

Using the same residual CNN architecture composed by 5 convolutional layers with batch normalisation and ReLU activations for the regularisation step, we explored the impact of using iteration-dependent networks. As the noise level varies when the number of iteration increases, using iteration-dependent networks allows having a customised regularisation for every iteration. Moreover, in order to ensure that the output of every module throughout the reconstruction of low-count data matches the output of the corresponding iteration of high-count data reconstruction using the OSEM algorithm, we introduce iteration-dependent targets and losses. Consequently, when 60 modules are unrolled, 60 different MSE loss terms monitor the training of the network. For FBSEM-Net and FBSEM-IS-Net, 10 iterations and 6 subsets (i.e. 60 modules) were used in order to have a similar number of iterations used as the one used to reconstruct images in routine scans.

**Fig. 1:**
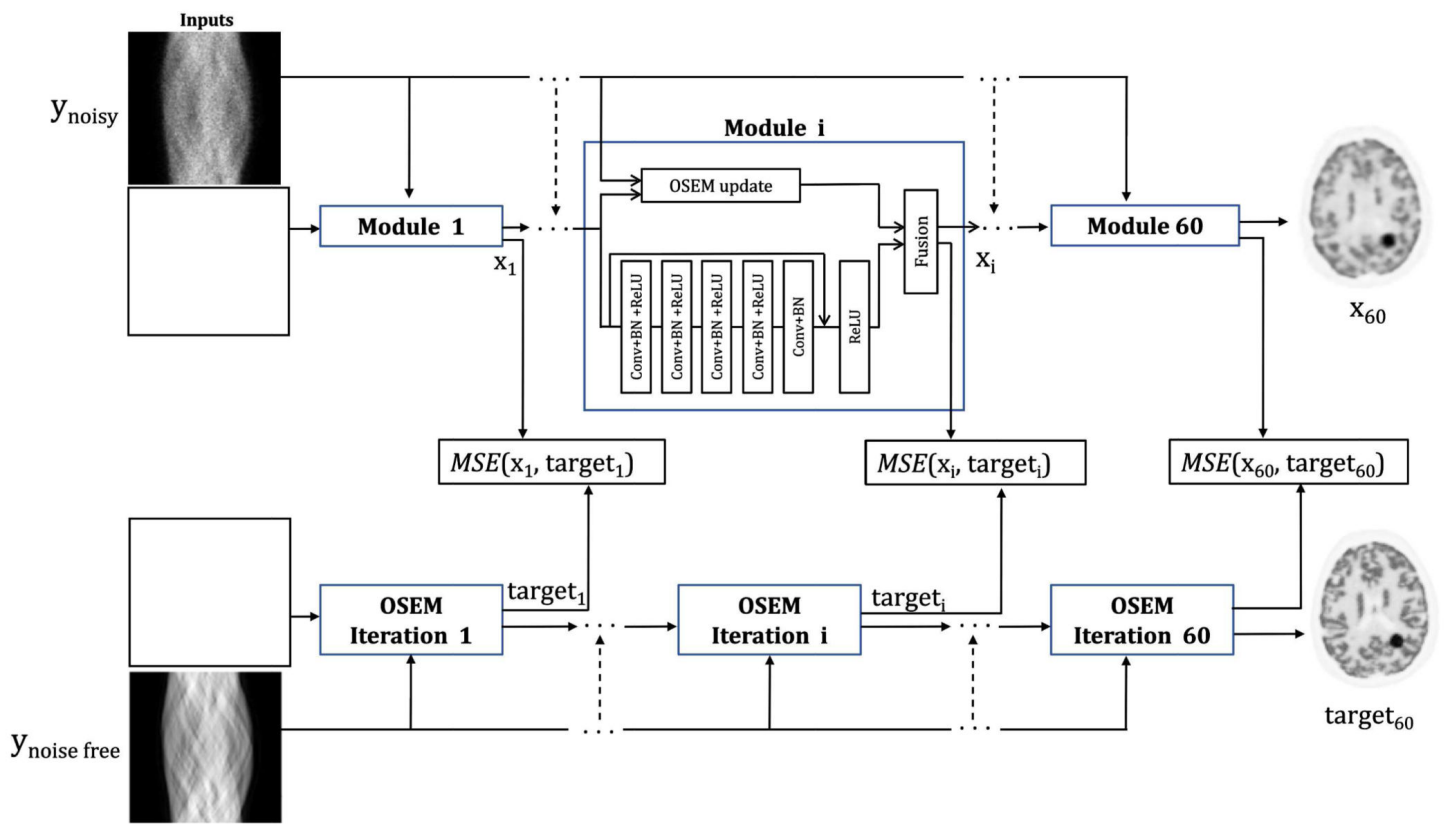
Overview of FBSEM-IS-Net. In this framework the regularisation network parameters ***θ**^n^* are iteration-dependent. The output of every module is compared to the corresponding output of the OSEM reconstruction of high-count data. In the original FBSEM-Net, only one final loss is used and the parameters of the regularisation networks are shared across all the modules. The blank squares represent the initial uniform image. CONV = Convolutional layer with 32 kernels of size 3 × 3, BN = batch normalisation, MSE = mean square error.

The global architecture of FBSEM-IS-Net is shown in Fig. 1. Furthermore, because the training of deep unrolled networks has massive memory requirements (especially for 3D data), we investigated the impact of training every module independently (or sequentially) rather than all at once (end-to-end).

## III Experimental Set-up

### A 2D datasets

FBSEM-Net is trained to map low-count sinograms to high-count reference PET images (Fig. 1). Twenty brain phantoms from BrainWeb were used to simulate 2D [^18^F]FDG PET images acquired with a Siemens Biograph mMR with a resolution of 2.08×2.08 mm^2^. Circular hot lesions with random radii in 2-8 mm and random locations were introduced in the PET phantoms. Attenuation, normalisation and image-space point-spread-function (PSF) modelling have been performed, although for simplicity randoms and scatters were not modelled. Five non-contiguous slices were selected from each phantom. For each sample, high quality PET images were reconstructed from the simulated measured data using the OSEM algorithm (*N_iterations_* = 10, *N_subsets_* = 6 and PSF with 2.5 mm full-width-at-half-maximum (FWHM) Gaussian kernels). These high-count (100M) reconstructions were used as targets. Low-count PET measured data (500k) were simulated from the original phantoms by introducing Poisson noise. For training, 80 samples were used while 10 samples were used for both validation and testing.

### B 3D datasets

T1-weighted MPRAGE MR images of 11 epilepsy and dementia patients collected at St Thomas' Hospital in London were used to generate realistic brain PET-MR phantoms. The process to simulate PET 3D images from real MR volumes was the following: i) segmentation into grey matter (GM), white matter (WM), cerebrospinal fluid (CSF), skull and skin using the SPM12 software, ii) assignment of random uptake values of 96.0 ± 5.0 and 32.0 ± 5.0 (arbitrary units) to GM and WM regions with a ratio of 3:1 between GM and WM and iii) insertion of spherical lesions with random radii in-between 2-8 mm and random locations. An attenuation map was generated by assigning attenuation values of 0.13, 0.0975 and 0 cm^−1^ to skull, tissues and air. The shape and voxel sizes from the original MR images were 230×230×254 and 1.04×1.04×1.01 mm^3^. The PET and attenuation maps were resized and resampled into the shape and voxel sizes of the standard PET images from a Siemens mMR scanner i.e. 344×344×127 and 2.08×2.08×2.03 mm^3^. All the images obtained were then rotated in the axial direction by 5 random angles within [0, 10] degrees, leading to 55 3D volumes. Once the phantoms were ready, noisy sinograms were generated, using PSF modelling in the forward model, attenuation, normalisation and Poisson noise, while again random and scatter coincidences were not modelled. Each sinogram had a matrix size of 344×252×837, identical to the standard sinogram format of the Siemens mMR scanner. The training set was composed of 45 samples, both the validation and testing set were composed of 5 samples.

### C Implementation

The network has been implemented in PyTorch and the training accelerated using a Nvidia Quadro k6000 12GB GPU. The data-consistency modules were implemented in Python using APIRL GPU-enabled PET projectors. The optimiser selected was the Adam optimiser with a learning rate of 0.01 for 50 epochs and a mini-batch size of 5 for 2D data and with a learning rate of 0.005 for 100 epochs with a mini-batch size of 1 for 3D data. The training was supervised using mean square error (MSE) losses.

### D Reference methods

To compare the different methods’ performance, the networks have been trained 3 times. Their performance was assessed with the MSE and the normalized root mean square error (NRMSE) defined as follows: $\mathrm{NRMSE}=\frac{\mathrm{RMSE}}{\bar{y}},$ with $\bar{y}$ the mean of the target dataset. In the original paper, FBSEM-Net has been compared to state-of-the-art reconstruction and post-reconstruction methods to demonstrate its performance. We compared here FBSEM-IS-Net trained with and without sequential training with various versions of FBSEM-Net. First, the original version from [4], then the original FBSEM-Net using iteration-dependent networks. We also trained a variant that we call FBSEM-Net leapfrogging, where the only difference with the original version is the target used for training. Rather than using the same number of modules as iterations to reconstruct the target, we trained FBSEM-Net leapfrogging to match the 60th iteration of the reconstruction of noise free data independently of the number of unrolled modules used. Therefore, FBSEM-Net leapfrogging matches the original FBSEM-Net only when 60 modules are used. We also compared the variants of FBSEM-Net with two conventional methods, the OSEM algorithm (*N_iterations_* = 10, *N_subsets_* = 6 with no PSF modelling and PSF with 4mm FWHM kernels), and the MAP-EM algorithm with a Tikhonov prior. The regularisation strength was optimised based on the MSE criterion. Finally, we compared our method with an unrolled method based on the INN from Lim et al. The main differences with the version proposed in [13] are that the input and output of the CED modules are neither normalised nor rescaled. Moreover, rather than using a ground truth image as target to train the networks (which does not exist for real data), high-count data reconstructions were used. We compared two different regulariser architectures. First, the CED proposed in the original paper consisting of an encoding convolutional layer with 78 kernels of size 3×3 (3×3×3 for 3D data), followed by a soft-thresholding operator $𝒯\left(x_{j},\alpha_{j}\right)=\mathrm{sign}\left(x_{j}\right)\;\text{max}\left(\left|x_{j}\right|-e^{-\alpha_{j}},0\right)$ with *α_j_* initialised at 15 and learned during the training and a decoding convolutional layer with 78 kernels each of size 3×3 (3×3×3 for 3D data). Then, the same residual CNN used in FBSEM-Net consisting of 5 convolutional layers with ReLU activations and batch normalisation. The two methods are hereafter referred to as INN CED and INN ResCNN. In this framework, an adaptive learning rate is used, nonetheless the user still has to set its strength (*c* = 0.05 here). Based on our observations, setting a wrong value for *c* is not compensated for by the adaptive learning rate *β*, it can therefore significantly impact the reconstructions. The pseudo-code of the INN used can be found in Algorithm 2. Table II clarifies the main differences between the reference methods and FBSEM-IS-Net.

**Algorithm 2: INN CED based on [13]**

**Initialize:**

  $\left\{{\text{c}}_{k}^{\left(n\right)},{\text{d}}_{k}^{\left(n\right)},\alpha_{k}^{\left(n\right)}:n=1\ldots N_{iters}\right\},c=0.05,K=78$

x^0^ obtained using 10 iterations of EM algorithm

**for** *n* = *1* … *N_iters_* **do**

      ${\text{x}}_{Reg}^{\left(n+1\right)}=\sum_{k=1}^{K}{\text{d}}_{k}^{\left(n+1\right)}*T({\text{c}}_{k}^{\left(n+1\right)}*\text{x}(n),\alpha_{k}^{\left(n\right)}$

      $\beta^{\left(n+1\right)}=c.\frac{{\left\|\text{s}-{\text{P}}^{T}\frac{\text{y}}{{\text{Px}}^{\left(n\right)}+\text{r}+\text{s}}\right\|}_{2}}{{\left\|{\text{x}}^{\left(n\right)}-{\text{x}}_{Reg}^{\left(n+1\right)}\right\|}_{2}}$

      with $s_{j}=\sum_{i}p_{ij}$

      $\lambda_{j}=\frac{1}{2}(s_{j}=\beta^{\left(n+1\right)}x_{j,Reg}^{\left(n+1\right)}$

      $V_{j}=x_{j}^{\left(n\right)}\sum_{i=1}^{M}p_{ij}\frac{y_{i}}{\sum_{j=1}^{N}p_{ij}x_{j}^{\left(n\right)}+r_{i}+s_{i}}$

      $x_{j}^{\left(n+1\right)}=\left\{ \begin{array}{l} \frac{\sqrt{\lambda_{j}^{2}+\beta^{\left(n+1\right)}V_{j}-\lambda_{j}}}{\beta^{\left(n+1\right)}}\;\text{if}\;\lambda_{j}<0\\ \frac{V_{j}}{\sqrt{\lambda_{j}^{2}+\beta^{\left(n+1\right)}V+\lambda_{j}}}\;\text{if}\;\lambda_{j}\geq 0 \end{array}\right.$

**end**

## IV Results

### A 2D datasets

#### 1) Impact of iteration-dependent networks and iteration-dependent targets

**Fig. 2:**
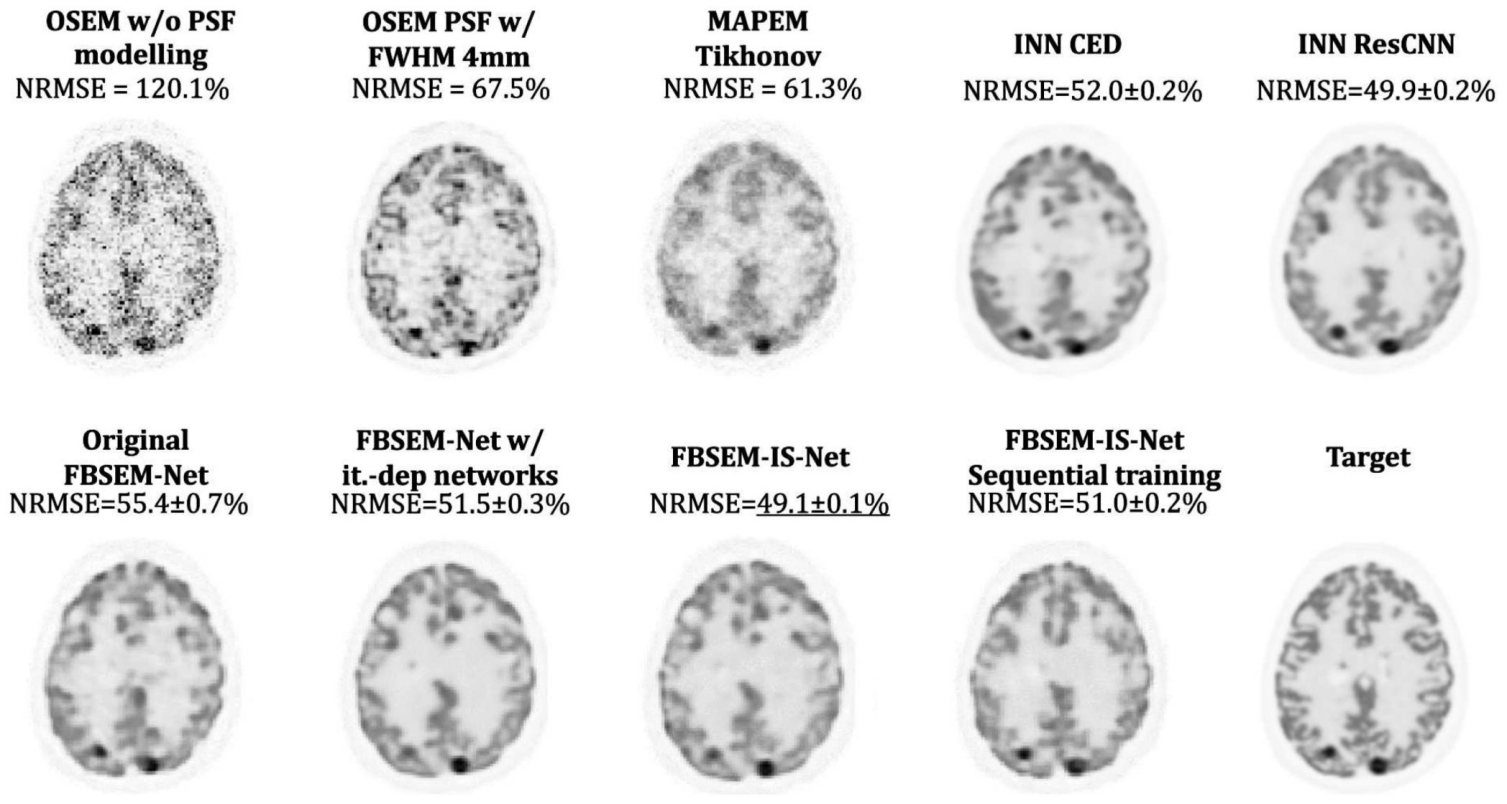
Comparison of the reconstructed images obtained using conventional methods (OSEM and MAP-EM with Tikhonov prior) and the various versions of FBSEM-Net and the INN on test data. Qualitatively, new versions of FBSEM-Net produced sharper reconstructions, quantitatively FBSEM-IS-Net is the best method in terms of NRMSE.

**Fig. 3:**
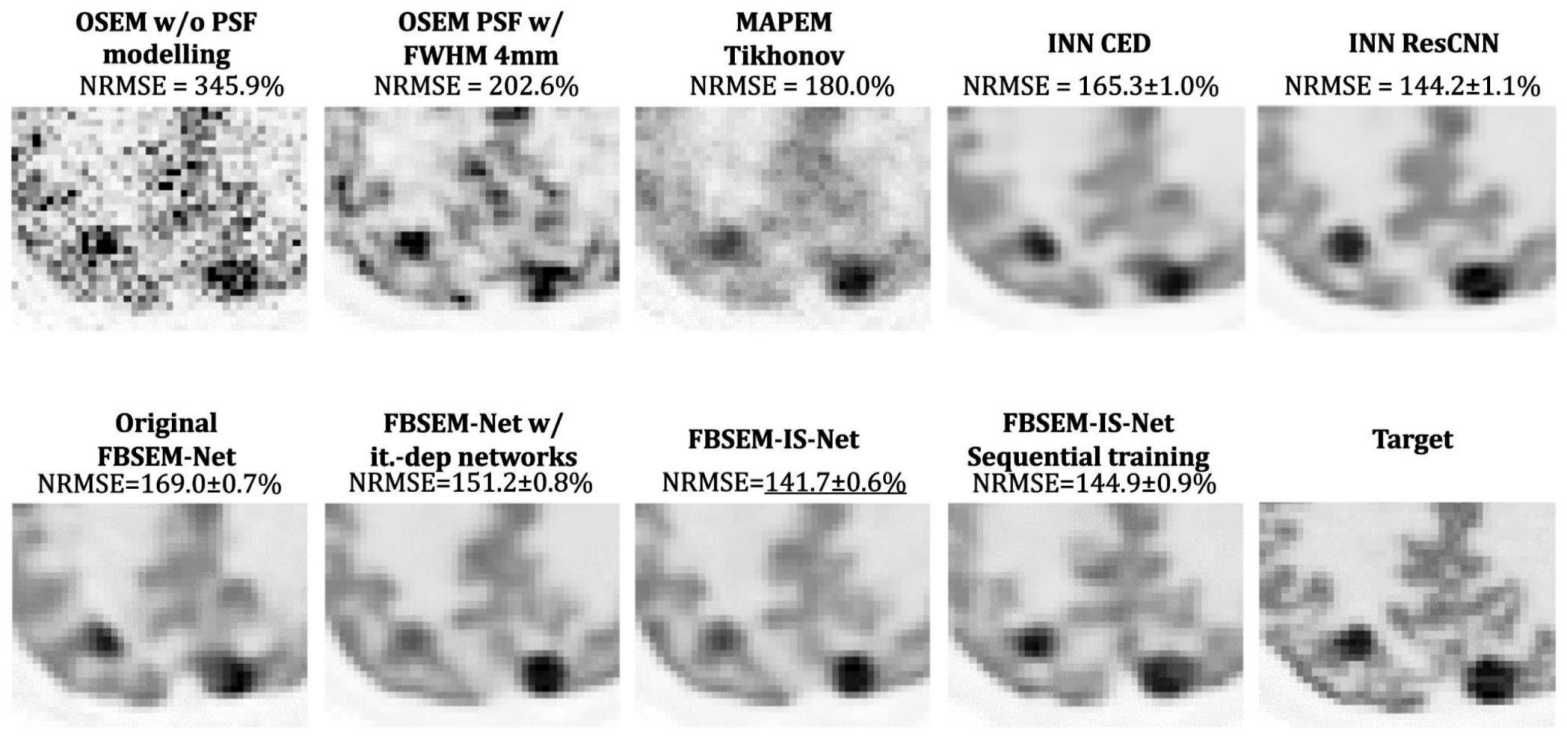
Zoom on the occipital lobe where two lesions are present. Deep learned methods often struggle to reconstruct smaller lesions. INN ResCNN and FBSEM-IS-Net are the two methods capable of correctly reconstructing the shape of lesions.

**Fig. 4:**
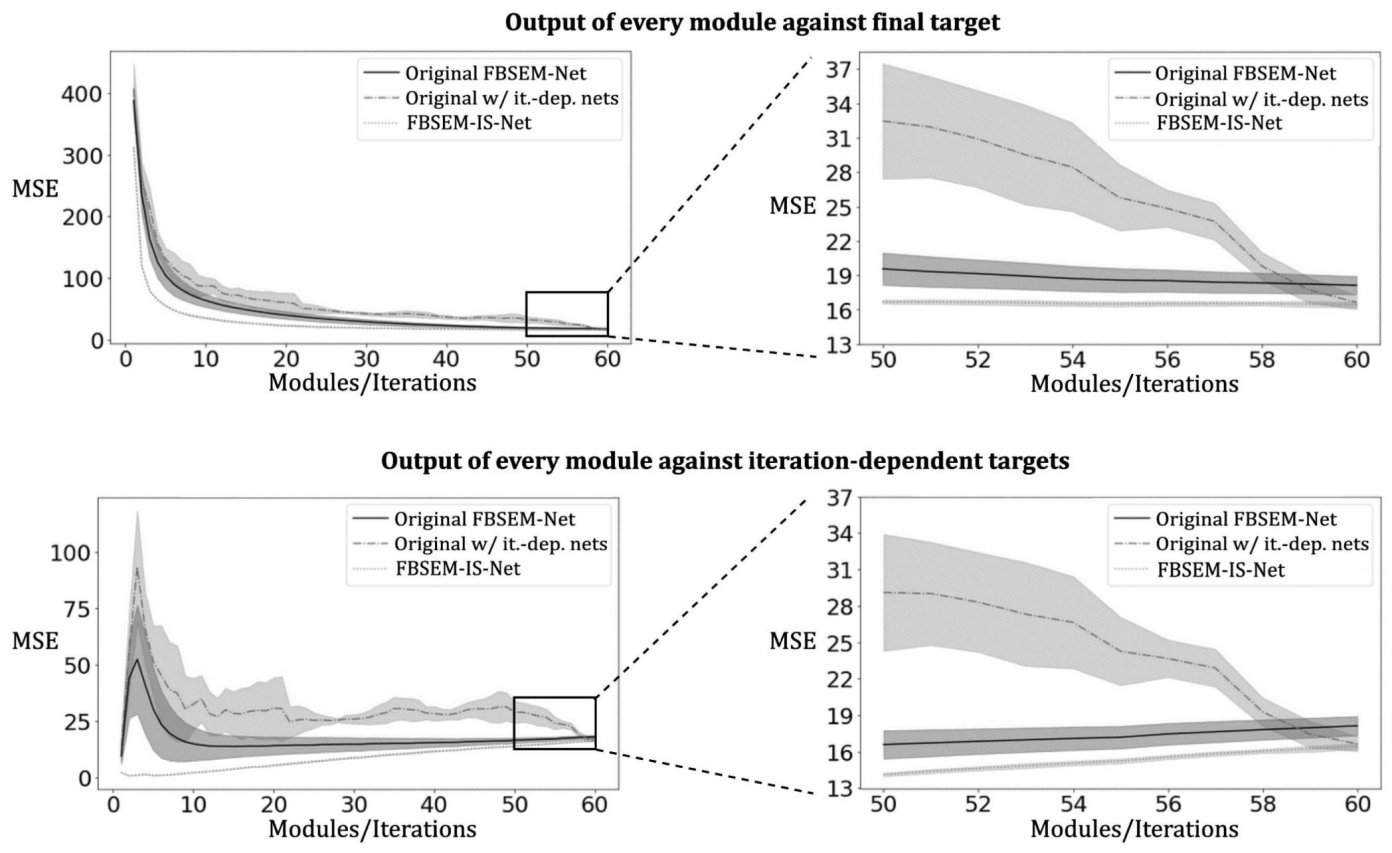
Top row: MSE computed between every module (i.e. iteration) output of the network and the final target (the final iteration from the high quality dataset). As expected, the loss decreases when the number of modules becomes closer to the number of iterations used to reconstruct the targets. The shaded area show the standard deviation of the loss across multiple training runs. Bottom row: MSE computed between every module (i.e. iteration) output and its own iteration-dependent target. The loss increases with the number of iterations as it becomes harder for the network to match more detailed images. The shaded area show the standard deviation of the loss across multiple training runs.

**Fig. 5:**
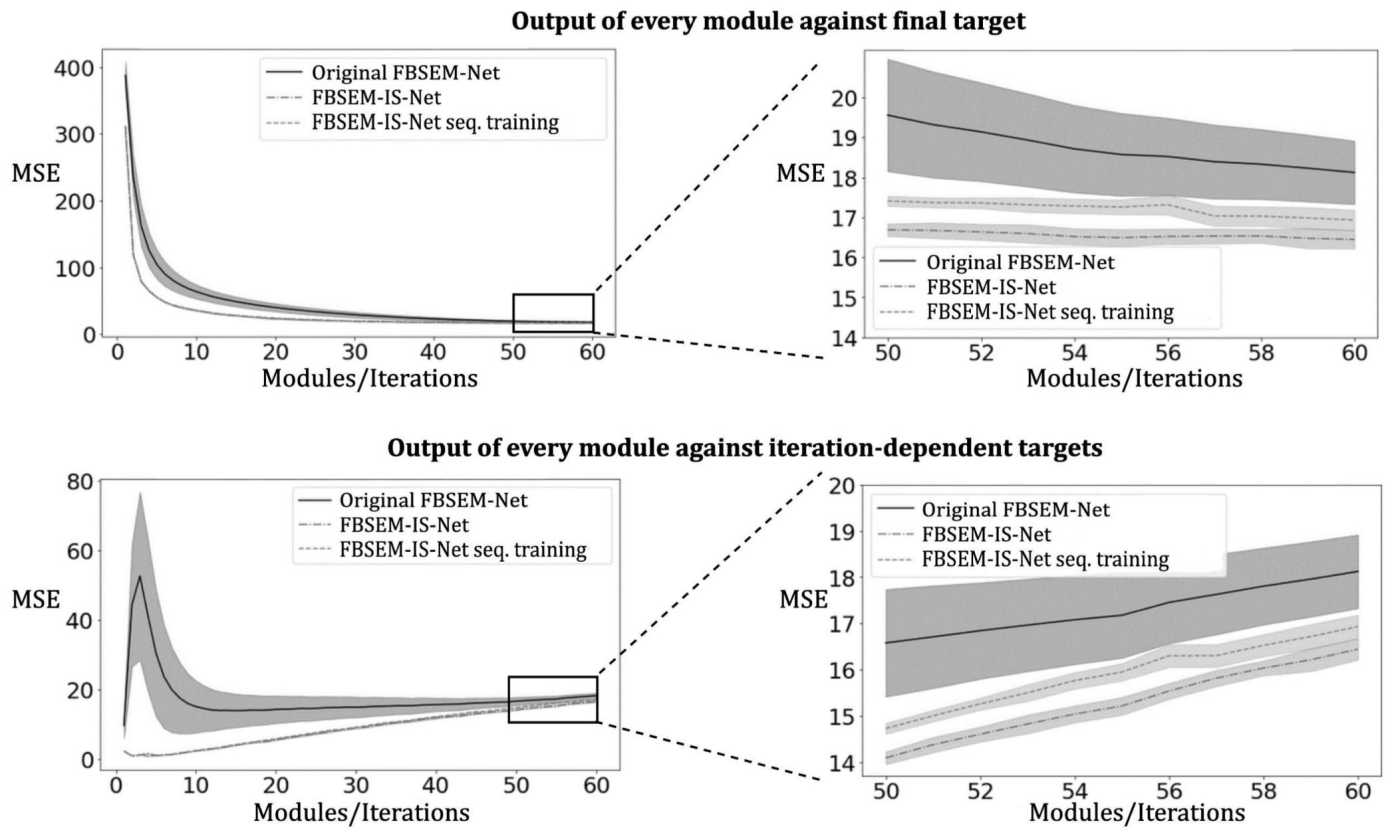
Top row: MSE computed between every module output of the network and the final target (the final iteration from the high quality dataset). Bottom row: MSE computed between every module output and its own iteration-dependent target. The shaded area show the standard deviation of the loss across multiple training runs.

The MSE between the output of every module and the target (Fig. 2, 3 and 4 top row) show that the use of iteration-dependent networks only or combined with iteration-dependent targets allows reduction of the MSE compared to the original FBSEM-Net. Quantitatively, FBSEM-IS-Net outperforms all the other methods both globally and locally. Qualitatively, the new versions offer sharper images compared to the original FBSEM-Net or the two INNs. While for the original FBSEM-Net the shape of both lesions is affected, with FBSEM-Net with iteration-dependent networks only the smallest lesion is not accurately reconstructed. In the case of FBSEM-IS-Net the correct shapes are preserved. Although, it can be noticed that the version with sequential training slightly underestimates the uptake in the right area of the lesion. This observation has been recurrent while using unrolled methods, the shape and/or the uptake of the lesions are often altered during the reconstruction. Fig. 4 shows that using iteration-dependent targets notably reduces the variance across multiple training runs. The shaded areas designating the standard deviation of the loss across multiple training runs, it can be noted that FBSEM-IS-Net is steady for each iteration as opposed to the other networks. Training at the module-level improves the consistency in the optimisation of the network parameters. The MSE between the output of every module and the corresponding OSEM iteration (Fig. 4 bottom row) may indicate that using iteration-dependent targets limits the network to only compensate for noise so that the entire MAPEM reconstruction of noisy data matches the reconstruction of noise free data. The original FBSEM-Net and the version with iteration-dependent networks only exhibit an erratic behavior for intermediate modules (from module 1 to 30 for the original version and 1 to 55 for the original version with iterationdependent networks). These networks solely learn to match the last module output to the final target. Therefore, one would not be able to use a lower number of modules at test time compared to what was used for training. In contrast, with FBSEM-IS-Net being trained iteration-wise, it becomes possible to train it once for a large number of iterations and use it for any lower or equal number of iterations. Using iteration-dependent targets imposes more control over the network training. In Fig. 4 and 5, the MSE increases with the number of iterations as high frequency noise amplification occurs at higher iterations and images with fine details become harder to reconstruct for the network. The output of neural networks, in particular when optimised with an MSE loss tend to produce smooth outputs. The use of this loss might as well explain the resulting affected lesion shapes. Adding a term to the loss function controlling the structure of the image, such as the structural similarity (SSIM), could potentially improve the shape of the lesions reconstructed.

#### 2) Impact of sequential training

Going from 2D to 3D real data with iteration-dependent networks and targets might raise memory issues during the training of such deep unrolled networks (Table I). A solution is to train the network sequentially i.e. one module at a time. As Fig. 2, 3 and 5 (bottom row) show, the impact of the sequential training is negligible both qualitatively and quantitatively. However, the reduced variability of the optimised network parameters from independent training runs is retained. The use of the sequential method compared to the conventional implementation allowed a reduction in memory usage from 224GB to 3.7GB resulting in a cut of about 98%. Nonetheless, time-consuming forward and back projectors are present in all the modules. Therefore, there is no computational time saving in the training process, as opposed to the INN framework, where the regularisers only are trained sequentially. Using the sequential training method proposed allows the training of deep unrolled networks that was not possible before due to hardware limitations. The memory requirements growing linearly with the number of modules, the original FBSEM-Net for 3D data cannot be trained using 60 modules. In the original paper, the authors set the number of modules to 12 (*N_iterations_* = 3, *N_subsets_* = 4) which uses the full capacity of a 12GB GPU. The sequential training enables the training of a fully unrolled FBSEM-IS-Net (60 modules) on any GPU. The possibility of using a higher number of unrolled modules thanks to sequential training also permits use of the typical number of iterations used for MAP-EM methods.

#### 3) Robustness to various distributions

**Fig. 6:**
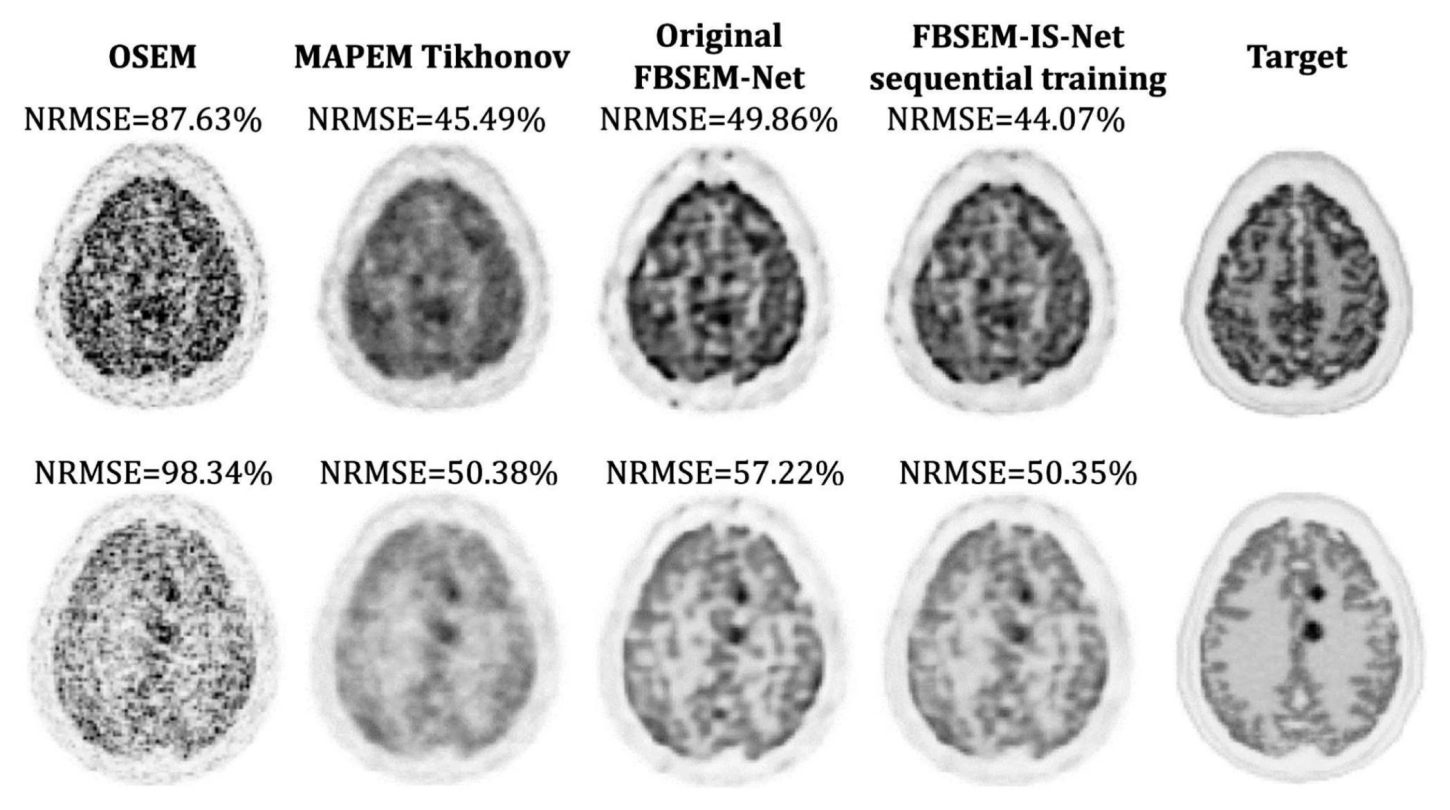
Reconstruction results for test dataset 1. The white matter intensity is higher than in the training set leading to higher noise levels in the white matter areas.

**Fig. 7:**
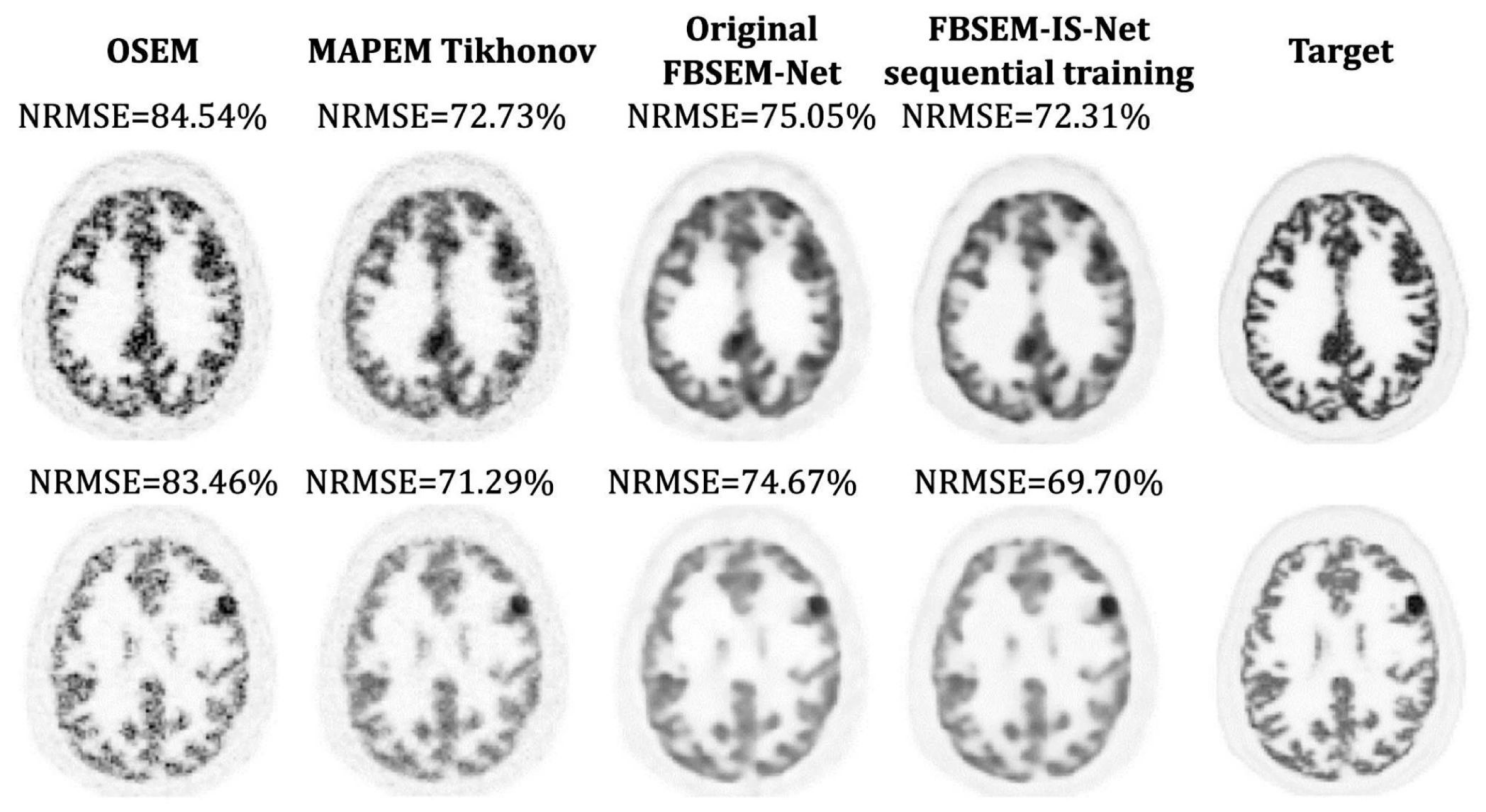
Reconstruction results for test dataset 2. The white matter intensity is lower than in the training dataset therefore noise is only present in the grey matter areas.

To demonstrate FBSEM-IS-Net generalisation abilities, two datasets with different radiotracer distributions than the data used for the network training have been simulated. The phantoms being piece-wise constant, we only varied the intensities for the grey matter, the white matter and the skin to generate new distributions. The various modifications can be found in Table III. For the first dataset, the white and grey matter intensity values were set to be more similar. This led to very noisy reconstructed images, the mean of the noise in the white matter being higher. For the second dataset, the opposite approach is considered by setting the white matter values low so as to increase the contrast between white and grey matter. The results obtained (Fig. 6 & 7) show that although the results are impacted by a change in distribution between training and testing, FBSEM-IS-Net is still outperforming the conventional methods. FBSEM performs better than MAP-EM with a Tikhonov prior, even though the regularisation parameter *β* has been fine-tuned specifically for the different distributions (*β* = 0.0650 for dataset 1, *β* = 0.0055 for dataset 2). For FBSEM-Net, no hyperparameter has to be set by the user, all were learned during the training phase. FBSEM-IS-Net also offers a reduction of the reconstruction error compared to the original version of FBSEM-Net in spite of having 60 times more trainable parameters. Using iteration-dependent targets appears to be enforcing the residual CNNs to remain regularisers only, without learning image details. Indeed, the output of every module only differ with its respective target due to noise therefore the network learns to account only for noise.

#### 4) Robustness to various noise levels

The original FBSEM-Net and the proposed version were tested for various noise levels. We used test data containing higher (1M) or lower counts (0.1M and 0.3M) compared to the measured data the networks were trained on (0.5M). While FBSEM-IS-Net performs better for 0.3M, 0.5M and 1M counts, the original FBSEM-Net is the best method for very low-count level. This might be justified by the low number of trainable parameters in this formulation (30k compared to 1.8M for the proposed version), which makes it more adaptive to very different noise levels at test time, but also by the limited number of training samples (80) in the dataset. Moreover, because the same regulariser is used for all the modules in the original FBSEM-Net and noise amplification is observed when the number of iterations increases, the residual CNN implicitly learns to deal with different reconstruction noises. With the proposed version, regularisation networks are iteration-dependent, trained for the reconstruction noise encountered at that iteration.[Fig F8]

#### 5) Robustness to multiple noise realisations

**Fig. 9:**
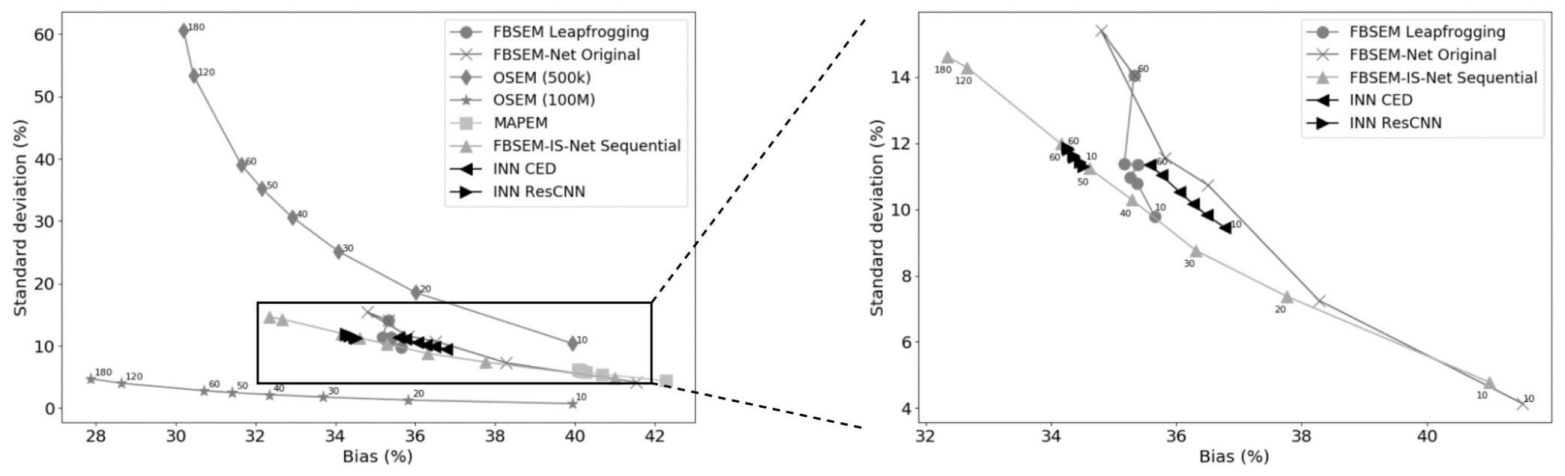
Bias-standard deviation trade-off curves. FBSEM-IS-Net is the method achieving the lowest bias when 60 modules or more are used. The markers designates the number of modules used. OSEM (100M) corresponds to the reconstruction of 50 noise realisations of high-count data and shows what ideally regularisation should achieve when used to reconstruct low-count data.

The various networks have been compared for 50 independent noise realisations using 0.5M counts. Here, for the sake of simplicity, all the networks have been trained twice only, however the uncertainties are not reported in Fig. 9 as their amplitudes were too low. To ensure a fair comparison, because no subsets are used in the INN, no subsets were used for the two FBSEM-Net versions and FBSEM-IS-Net to conduct the bias-standard deviation analysis. The reconstructed images were evaluated using the root mean square error (RMSE):

(10) $$RMSE=\sqrt{{\text{bias}}^{2}+SD^{2}}$$

with the bias defined by:

(11) $$\text{bias}=\sqrt{\frac{\sum_{j\in \Omega }{\left({\bar{x}}_{j}-x_{j}^{Ref}\right)}^{2}}{\sum_{j\in \Omega }x_{j}^{Ref}{}^{2}}}$$

and the standard deviation by:

(12) $$SD=\sqrt{\frac{1}{S}\frac{\sum_{s=1}^{S}\sum_{j\in \Omega }{\left({\bar{x}}_{j}-x_{j}^{\text{Ref}}\right)}^{2}}{\sum_{j\in \Omega }{\left(x_{j}^{Ref}\right)}^{2}}}$$

${\bar{x}}_{j}$ being the mean reconstructed value for voxel *j*, obtained by averaging the *S* = 50 realisations, *x^Ref^* the ground truth image (different to the target obtained by reconstruction of high-count data used to train the networks) and Ω the set of the image voxels. Various numbers of modules ranging from 10 to 60 for the unrolled methods without sequential training, 10 to 120 for the unrolled methods with sequential training and 10 to 180 for the OSEM and MAPEM algorithms were used. For every number of modules selected, a different network was trained, therefore each marker in figure 9 corresponds to the performance of a network trained independently. The results obtained show that FBSEM-IS-Net with sequential training achieved the lowest bias when using 60 modules or more. This trend becomes more significant as the number of modules increases. The performance of FBSEM-IS-Net without sequential training has not been reported in Fig. 9 as its curve and the one from FBSEM-IS-Net with sequential training were indistinguishable. As expected, when the number of modules increases, the bias decreases while the standard deviation increases. The gap between the original FBSEM-Net and FBSEM-IS-Net sequential training becomes wider with higher numbers of modules. The use of iteration-dependent targets and losses helps to reduce the variance of the network by constraining it to remain purely a denoiser. The two INNs along with FBSEM-Net leapfrogging are the methods the least impacted by a change in the number of modules. It appears that training all the regularisers with the same target allows for the performance to be more independent of the number of modules used. This effect has benefits if the number of modules were to be limited to conduct fast reconstructions for instance.

### B 3D datasets

**Fig. 10:**
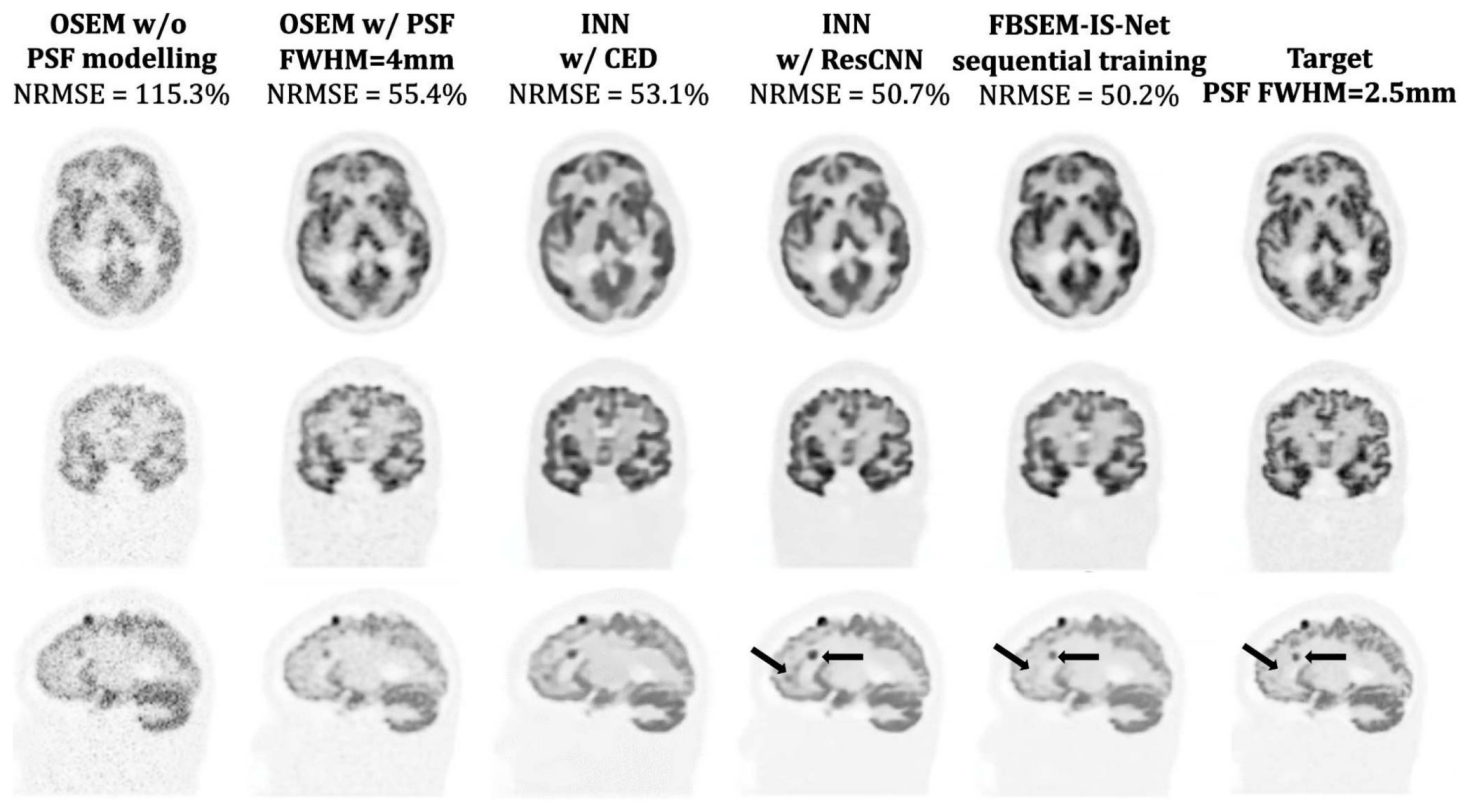
Reconstruction of 3D simulated data (from top to bottom: axial, coronal and sagittal views). The lesion in the image reconstructed using the iterative NN with the residual CNN regulariser seems bigger than in the target (right arrow). It can also be noticed that the two INNs add grey matter where there should be only white matter (left arrow).

Reconstruction results for 3D data show that FBSEM-IS-Net is the best method in terms of NRMSE. The INN using the residual CNN architecture has a similar performance to FBSEM-IS-Net, although the shape of the reconstructed lesion seems altered (Fig. 10). FBSEM-IS-Net offers sharper reconstruction. The output of the INN with the CED architecture is noisier than the ones from the INN ResCNN and FBSEM-IS-Net. The original FBSEM-Net has not been compared here with the other methods as it is not possible to train it for 3D data using 60 modules.

## V Conclusion

We have proposed a new version of the forward-backward splitting expectation-maximisation network [4] as well as a memory-efficient training method for deep unrolled networks. We demonstrated that using iteration-dependent networks permits a reduction of the reconstruction error compared to the original formulation of FBSEM-Net. We also showed that using iteration-dependent targets in FBSEM-IS-Net, intended to ensure that the network remains a regulariser only and does not learn object details, stabilises the training of the network by improving the consistency in the optimisation of the network parameters. FBSEM-IS-Net has been demonstrated to be stable when tested with test data with a different radiotracer distribution than the training data, with multiple noise realisations as well as various noise levels, attesting to good generalisation abilities in spite of having a high number of trainable parameters. Lastly, we showed that training all the modules sequentially rather than in an end-to-end fashion drastically reduces the memory requirements. It gives the possibility to train deeper unrolled networks with 2D but especially 3D data, without notably affecting the performance of the network. The proposed method allows a correct iteration by iteration unrolling of the MAP-EM algorithm, which was not previously feasible when end-to-end training was performed. While only demonstrated for FBSEM-Net, the use of iteration-dependent targets/losses and sequential training can be applied to any unrolled method.

## VI Discussion

FBSEM-IS-Net has been shown to be robust for different piece-wise constant distributions despite having 60 times more trainable parameters than FBSEM-Net. Nonetheless, a more comprehensive assessment of the proposed network's generalisation abilities is still needed. Future work will focus on further demonstrating the abilities of unrolled networks trained with iteration-dependent networks and targets to generalise better to different distributions than unrolled methods with only iteration-dependent networks or with one regularisation network whose parameters are shared across all the modules. Ideally, the impact of removing some of the backpropagation of the errors by training the modules sequentially should be theoretically investigated. When all the modules are trained together, the errors are backpropagated from the last module to the first one, whereas in the sequential training framework, the final modules regularisers errors do not impact the training of the previous ones. Ultimately, various loss functions should be explored in order to assess their capabilities for accurate reconstruction of lesions shapes and uptake.

## Figures and Tables

**Fig. 8 F8:**
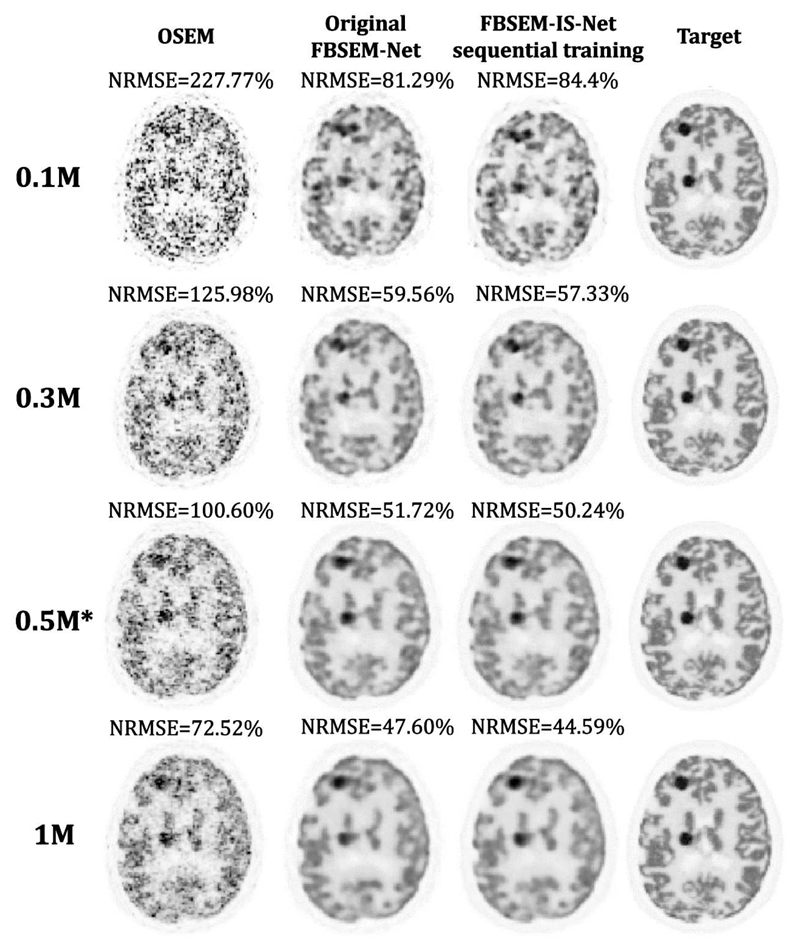
Reconstruction results for various noise levels. FBSEM-Net and FBSEM-IS-Net were trained using 0.5M count measured data.

**Table I:** Comparison of Unrolled pet reconstruction methods

Name	Architecture [total trainable parameters]	No. unrolled modules	Iteration-dependent networks	Iteration-dependent targets	Leapfrogging	Sequential Training	Memory requirements for training (mini-batch size=l, image size 172×172×127)
**EM-Net** Gong et al. [19]	U-Net: 3 encoding stages (2C+BN+LReLU), 3 decoding stages (2C+BN+LReLU), 2C+BN+LReLU at bottleneck, skip connections (add) instead of concatenating [~2 million parameters]	10	No	No	Yes	No	12.0 GB
**MAPEM-Net** Gong et al. [12]	U-Net: 3 encoding stages (2C+BN+LReLU), 3 decoding stages (2C+BN+LReLU), 2C+BN+LReLU at bottleneck, skip connections (add) instead of concatenating [~(8×2)=16 million parameters]	8	Yes	No	Yes	No	12.0 GB
**INN** Lim et al. [13]	Convolutional autoencoders, each composed of 2 C layers and soft thresholding operator in between [~(10×4000) = 40,000 parameters]	10	Yes	No	Yes	Yes	4.5 GB
**FBSEM-Net** Mehranian and Reader [4]	Residual CNN: 5C layers each with BN+ReLU [~80,000 parameters]	60	No	No	No	No	222.0 GB
**FBSEM-IS-Net**	Residual CNN: 5C layers each with BN+ReLU [~(60×80000)= 4800000 parameters]	60	Yes	Yes	No	No	224.0 GB
**FBSEM-IS-Net with sequential training**	Residual CNN: 5C layers each with BN+ReLU [~(60×80000)= 4800000 parameters]	60	Yes	Yes	No	Yes	3.7 GB

* C = Convolutional layer, BN = Batch normalisation, LReLU = Leaky ReLU

**Table II:** Comparison Of The Proposed And Reference Deep Learned Methods

Name	No. of targets	Target image	Leapfrogging factor*	Regularisation hyperparameter	No. regularisers	Architecture	Training	Initialisation
**FBSEM-Net original version**	1	Last iteration of noise free data reconstruction	1	Learned	1	Residual CNN	End-to-end	Uniform image
**FBSEM-Net leapfrogging**	1	Last iteration of noise free data reconstruction	60/No. modules	Learned	1	Residual CNN	End-to-end	Uniform image
**FBSEM-IS-Net**	No. modules	Corresponding iteration of noise free data reconstruction	1	Learned	No. modules	Residual CNN	Sequential	Uniform image
**INN CED**	1	Last iteration of noise free data reconstruction	60/No. modules	Non learned	No. modules	Convolutional Encoder-Decorder	Sequential	10th iteration of EM reconstruction
**INN ResCNN**	1	Last iteration of noise free data reconstruction	60/No. modules	Non learned	No. modules	Residual CNN	Sequential	10th iteration of EM reconstruction

* Leapfrogging factor: If the number of modules used in the unrolled network is equal to the number of iterations used to reconstruct the targets, the factor equals to 1. When 10 modules are used to match a target reconstructed with 60 iterations, the factor equals 60/10.

**Table III:** Intensity values set for the different distributions

	Training dataset	Test dataset 1	Test dataset 2
**White matter**	32	64	5
**Grey matter**	96	80	120
**Skin**	16	32	32
